# Editorial: Clinical, biological, and economic aspects of pediatric infections in Latin America

**DOI:** 10.3389/fpubh.2024.1398071

**Published:** 2024-03-22

**Authors:** Juan Garduño-Espinosa, Fortino Solórzano-Santos, Guillermo Salinas-Escudero, Guadalupe Miranda-Novales, Joaquin F. Mould-Quevedo, Diana Avila-Montiel

**Affiliations:** ^1^Division of Research, Hospital Infantil de México Federico Gómez, Mexico City, Mexico; ^2^Infectious Diseases Research Department, Hospital Infantil de México Federico, Mexico City, Mexico; ^3^Center for Economic and Social Studies in Health, Hospital Infantil de México Federico Gómez, Mexico City, Mexico; ^4^Analysis and Synthesis of Evidence Research Unit, XXI Century National Medical Center, Mexican Social Security Institute, Mexico City, Mexico; ^5^CSL Seqirus USA Inc., Summit, NJ, United States

**Keywords:** infectious diseases, children, pediatric, health economics, Latin America

In Latin American countries, there is a diversity of infectious diseases that include tropical diseases, diseases related to poverty and malnutrition, and those neglected diseases related to inadequate sanitation, with close contact with infectious vectors and domestic animals. As a consequence, the exaggerated use of antimicrobials has also had an impact on the increase in bacterial resistance (1).

Among the neglected tropical diseases, parasitic diseases, dengue, malaria, and Zika occupy the first places in the Central American region and Mexico, despite a decrease in the frequency of some of these diseases (2). In the group of neglected diseases in children under 5 years of age, the most common disease is malaria, followed by parasitic infections and dengue (3).

Infectious diseases can be life-threatening in children and can also leave lifelong consequences by affecting their growth and neurodevelopment (4).

Addressing infectious diseases in this region is complex due to various determinants such as climate variability, political and security problems, such as violence and organized crime, inequity in the distribution of resources, migration, and food insecurity (5, 6).

The pediatric population is especially vulnerable to these determinants, so it is essential to generate evidence to carry out intervention strategies from a clinical, biological, social, and economic perspective to improve access to health systems and the management of infectious diseases.

This Research Topic consists of four original articles. It compiles evidence and experiences in managing infectious diseases from some Latin American countries, showing data on the pediatric populations of these countries, in which multiple social and cultural aspects are shared.

Below are the topics addressed in the articles contained in this Research Topic.

Bacteremia due to carbapenem-resistant enterobacterales (CRE) is a problem associated with medical care worldwide; in addition, the primary source of infection cannot be identified in most cases, so it is essential to carry out research that explores the different factors that produce it to identify them and be able to carry out infection prevention and control strategies (7–9). The study by Ruvinsky et al. in Argentina identified risk factors for acquiring bacteremia due to CRE in pediatric patients. Among the variables analyzed are demographic, epidemiological, clinical, microbiological, and resource management factors. These results show the importance of establishing comprehensive health programs and policies to improve the appropriate use of antimicrobials. Mortality associated with carbapenem resistance is a challenge worldwide (10).

In pediatric cancer patients, bloodstream infections continue to be a leading cause of morbidity and mortality, especially in low-income patients due to their living conditions and barriers to accessing medical care. Bloodstream infections have also been associated with increased hospitalization time and resource consumption (11–13).

A study in Guatemala by Mukkada et al. found that it is necessary to improve infection control practices and invest in laboratory diagnosis and data recording to identify and document factors contributing to poor outcomes. Modifying negative factors will allow for strategies that will impact the management of healthcare-associated infections, particularly in vulnerable settings. From the prevention approach, the effectiveness of hand hygiene for the prevention of hospital-acquired infections is widely known, and there are standardized protocols; however, adoption and adherence to these protocols continue to be a challenge (14, 15).

The study by Salinas-Escudero et al. in Mexico showed the importance of implementing Automated hand hygiene monitoring (AHHM) to promote hand hygiene and thereby prevent infectious complications. The study demonstrated that it is a profitable strategy since it effectively reduces costs and hospital infections.

Regarding emerging infectious diseases, it is known that COVID-19 represents an economic burden for health systems in the world and is a challenge for health authorities, especially in a country like Mexico, where the health system is fragmented (16, 17).

The study carried out in Mexico City by Reyes-López et al. estimated the direct medical costs associated with the treatment of children with COVID-19 in a tertiary care pediatric hospital. It took into account various factors in the patients to make accurate estimates of the care costs and thus carry out budgetary and input planning for their care.

This Research Topic outlines some of the transcendental factors that influence the care and management of infectious diseases in the pediatric population in a complex and heterogeneous region such as Latin America.

On the other hand, the influence of certain factors on clinical outcomes was explored in statistical models, identifying some that may even modify the outcome.

This Research Topic should be analyzed with the perspective that its content will contain descriptive texts and aspects most applicable to the region.

It is interesting to emphasize the importance of overcoming geographical barriers and inviting scientific collaboration to work to better address pediatric diseases in the region.

## Author contributions

JG-E: Conceptualization, Writing – original draft. FS-S: Writing – review & editing. GS-E: Writing – review & editing. GM-N: Writing – review & editing. JM-Q: Writing – review & editing. DA-M: Writing – review & editing.
